# Imatinib-Induced Recurrent Pleural Effusions

**DOI:** 10.7759/cureus.83153

**Published:** 2025-04-28

**Authors:** Pranali S Pachika, Ranadheer R Dande, Phuong Ngo

**Affiliations:** 1 Hematology and Oncology, University of Louisville, Louisville, USA; 2 Internal Medicine, Baptist Health Hardin Hospital, Elizabethtown, USA; 3 Medical Oncology/Hematology, James Graham Brown Cancer Center, University of Louisville School of Medicine, Louisville, USA

**Keywords:** dasatinib, gastrointestinal stromal tumor (gist), imatinib therapy, rare cause of pleural effusion, tyrosine kinase inhibitor (tki)

## Abstract

Imatinib, a tyrosine kinase inhibitor, is widely used for treating gastrointestinal stromal tumors (GISTs) and chronic myeloid leukemia (CML). While commonly associated with mild fluid retention, significant pleural effusion is an uncommon but potentially serious adverse effect. We present a case of recurrent pleural effusions secondary to imatinib therapy in a 62-year-old female patient with metastatic lung adenocarcinoma and a concurrent GIST harboring an exon 9 mutation. She was initiated on imatinib 400 mg daily, later increased to twice daily. Within weeks, she developed progressive dyspnea, and imaging revealed large bilateral pleural effusions. Pleural fluid analysis demonstrated an exudative effusion, with cytology and microbiological studies ruling out infection or malignancy. Cardiac function was preserved, and there were no signs of volume overload. She underwent multiple thoracenteses for symptomatic relief. Due to recurrent pleural effusions, imatinib was permanently discontinued, leading to complete resolution of the effusions. Subsequent treatment with sunitinib was not tolerated due to severe mucositis and cytopenias. Despite discontinuation of targeted therapy, both her GIST and metastatic lung cancer remained stable under surveillance. While pleural effusions are frequently reported with dasatinib, they are rare with imatinib. The proposed mechanisms include inhibition of platelet-derived growth factor receptors (PDGFRs), leading to increased vascular permeability, impaired lymphatic drainage, and renal sodium retention. Dose reduction may mitigate fluid retention; however, our patient developed significant pleural effusions at standard dosing, necessitating treatment discontinuation. This case underscores the importance of recognizing pleural effusion as a rare but serious adverse effect of imatinib therapy. Clinicians should maintain a high index of suspicion for drug-induced pleural effusions, particularly in the absence of other etiologies, and consider discontinuation if clinically indicated. Early recognition and management can prevent complications and improve patient outcomes.

## Introduction

Imatinib mesylate, a tyrosine kinase inhibitor (TKI), has revolutionized the treatment of chronic myeloid leukemia (CML) and gastrointestinal stromal tumors (GISTs) by targeting the BCR-ABL fusion protein and c-KIT mutations, respectively [1]. It was approved in 2001 for CML and in 2003 for GIST tumors. Since its approval, imatinib has significantly improved survival outcomes and quality of life for patients with these malignancies [2]. Despite its efficacy, imatinib is associated with a spectrum of adverse effects, including hematologic toxicities, hepatotoxicity, and fluid retention. Among the latter, pleural effusion is a recognized but relatively uncommon adverse event that warrants further clinical attention [2-4]. We report a case of a 62-year-old woman who developed recurrent pleural effusions following the initiation of imatinib therapy for a GIST, with no other identifiable cause. The effusion resolved after the medication was discontinued.

## Case presentation

The patient is a 62-year-old woman with a medical history significant for a 40-pack-year smoking history and estrogen receptor-positive ductal carcinoma in situ of the left breast, diagnosed in 2015 and treated with lumpectomy and radiation therapy, followed by adjuvant tamoxifen for five years. She initially presented to the oncology clinic after a low-dose chest computed tomography (CT) showed enlarged pulmonary lymph nodes. A subsequent PET/CT scan showed multiple fluorodeoxyglucose (FDG)-avid pulmonary nodules in both lungs, hilar lymphadenopathy, subcarinal lymphadenopathy, and a right retroperitoneal lymph node suggestive of metastatic disease. The biopsy of the subcarinal lymph node returned with adenocarcinoma consistent with lung primary. Additional tumor testing showed a PD-L1 score of 90% and no actionable mutations. She was treated with carboplatin, pemetrexed, and pembrolizumab, followed by maintenance pemetrexed and pembrolizumab; she stopped pemetrexed after 19 cycles due to fatigue and continued pembrolizumab maintenance alone.

**Figure 1:**
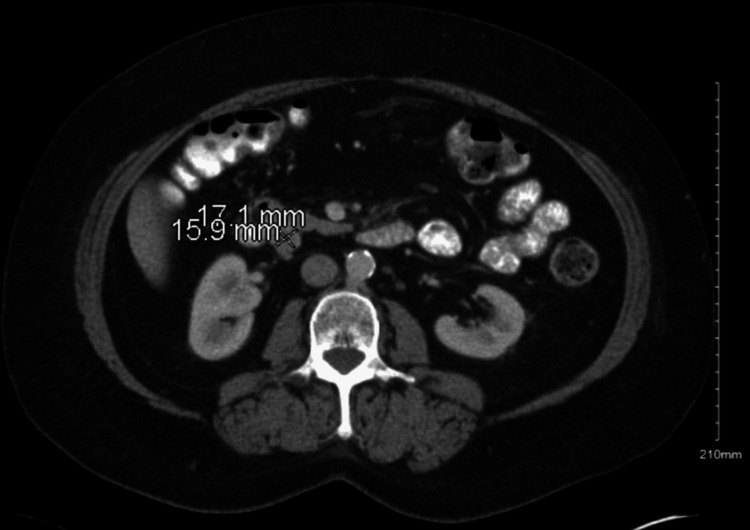
Computed tomography of the abdomen showing a mass in the posterior pancreaticoduodenal groove

Imaging showed treatment response for a year and a half until a repeat CT revealed a 1.7 cm soft tissue lesion in the posterior pancreaticoduodenal groove, as shown in Figure 1.

**Figure 2:**
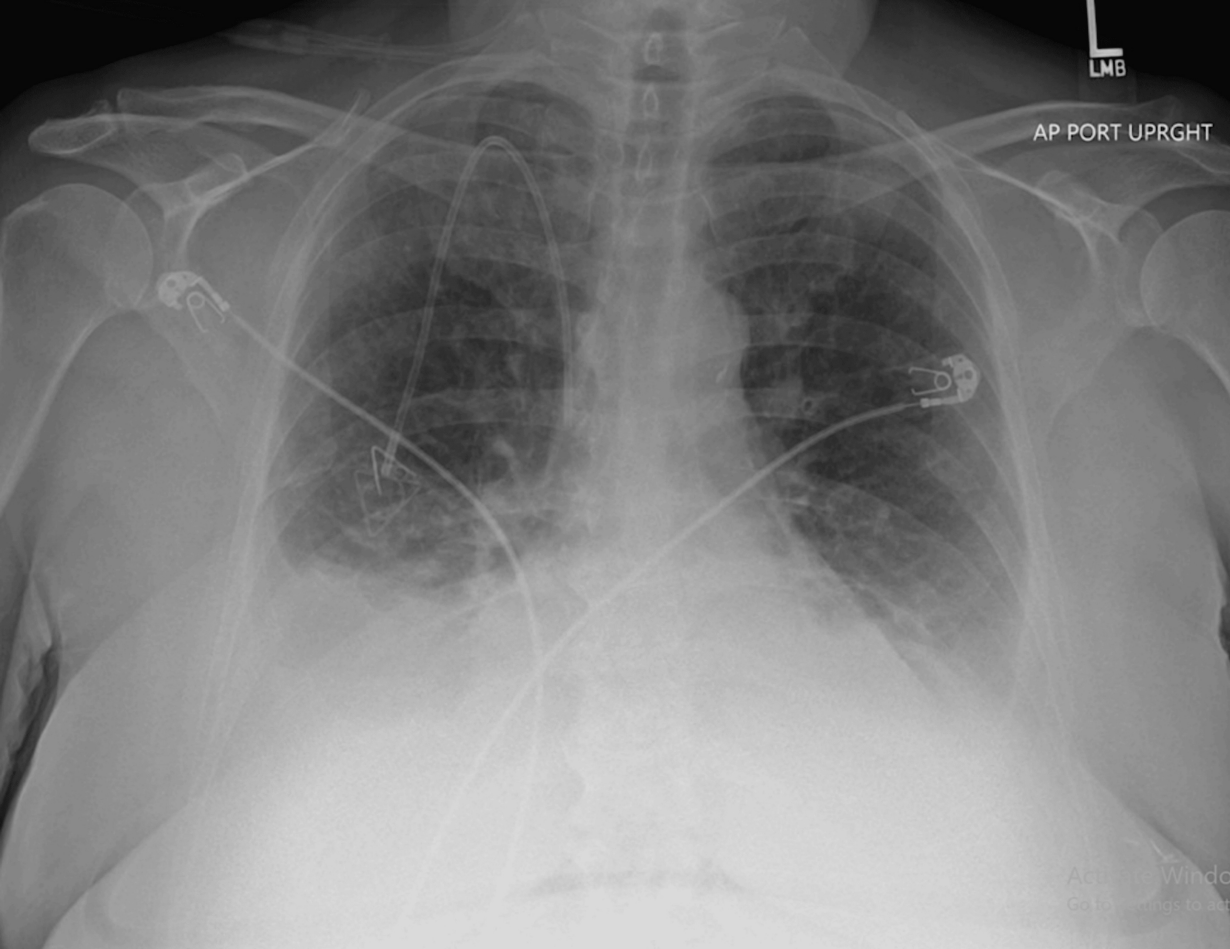
Chest X-ray showing bilateral pleural effusion

**Figure 3:**
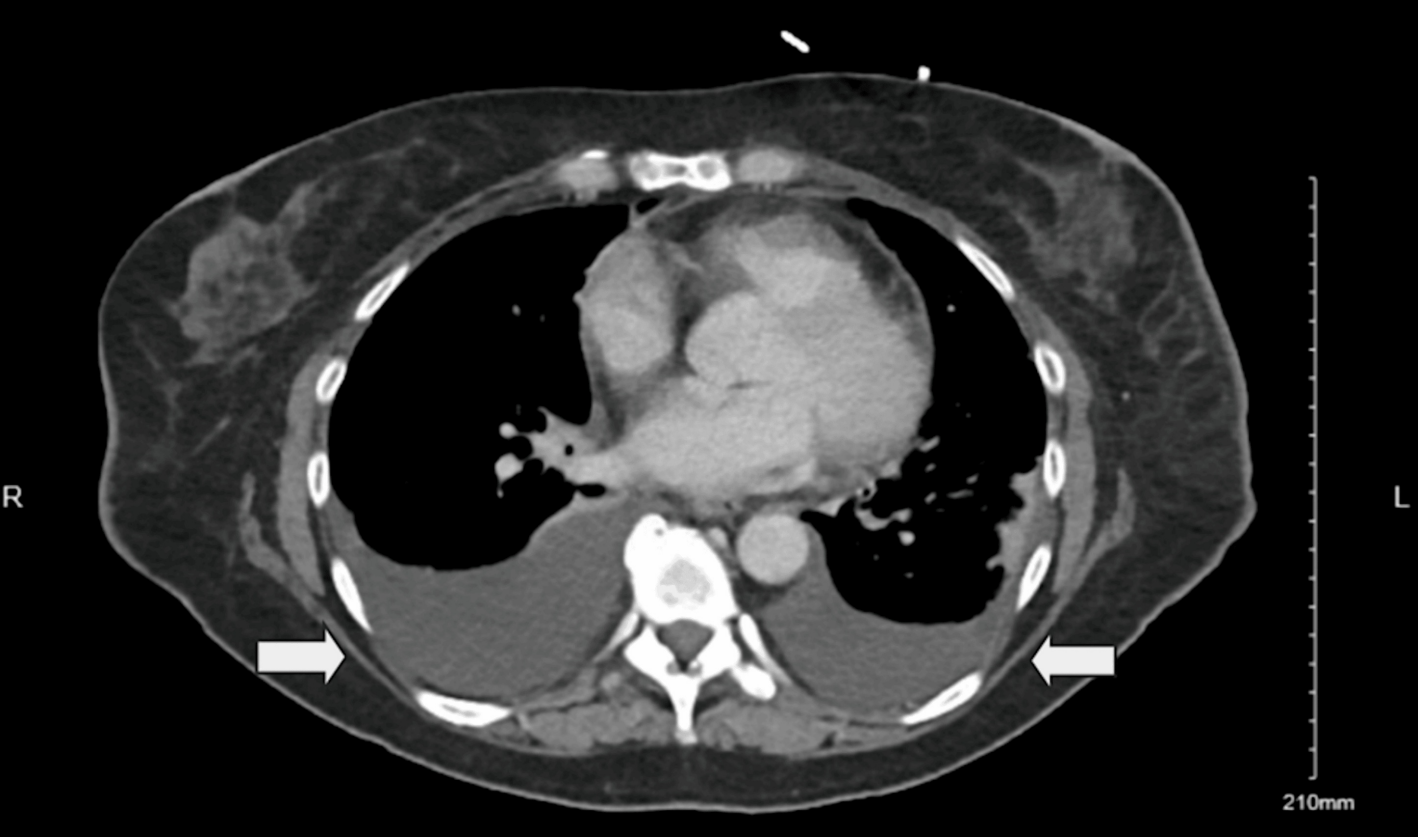
Chest computed tomography (CT) of the patient showing bilateral pleural effusion

Upon reviewing previous images, this lesion had been present but was presumed to be a metastatic lymph node. It remained stable in size, but she underwent an endoscopic ultrasound (EUS) and biopsy for further evaluation. Pathology results indicated a GIST with a Ki-67 index of 3%. Further genomic testing showed an exon 9 mutation, prompting the initiation of imatinib 400 mg, initially administered daily and then BID. A few weeks later, she developed acute shortness of breath. Figure 2 illustrates the evaluation conducted using a chest X-ray, and Figure 3 depicts a CT of the chest showing large bilateral pleural effusions.

**Figure 4:**
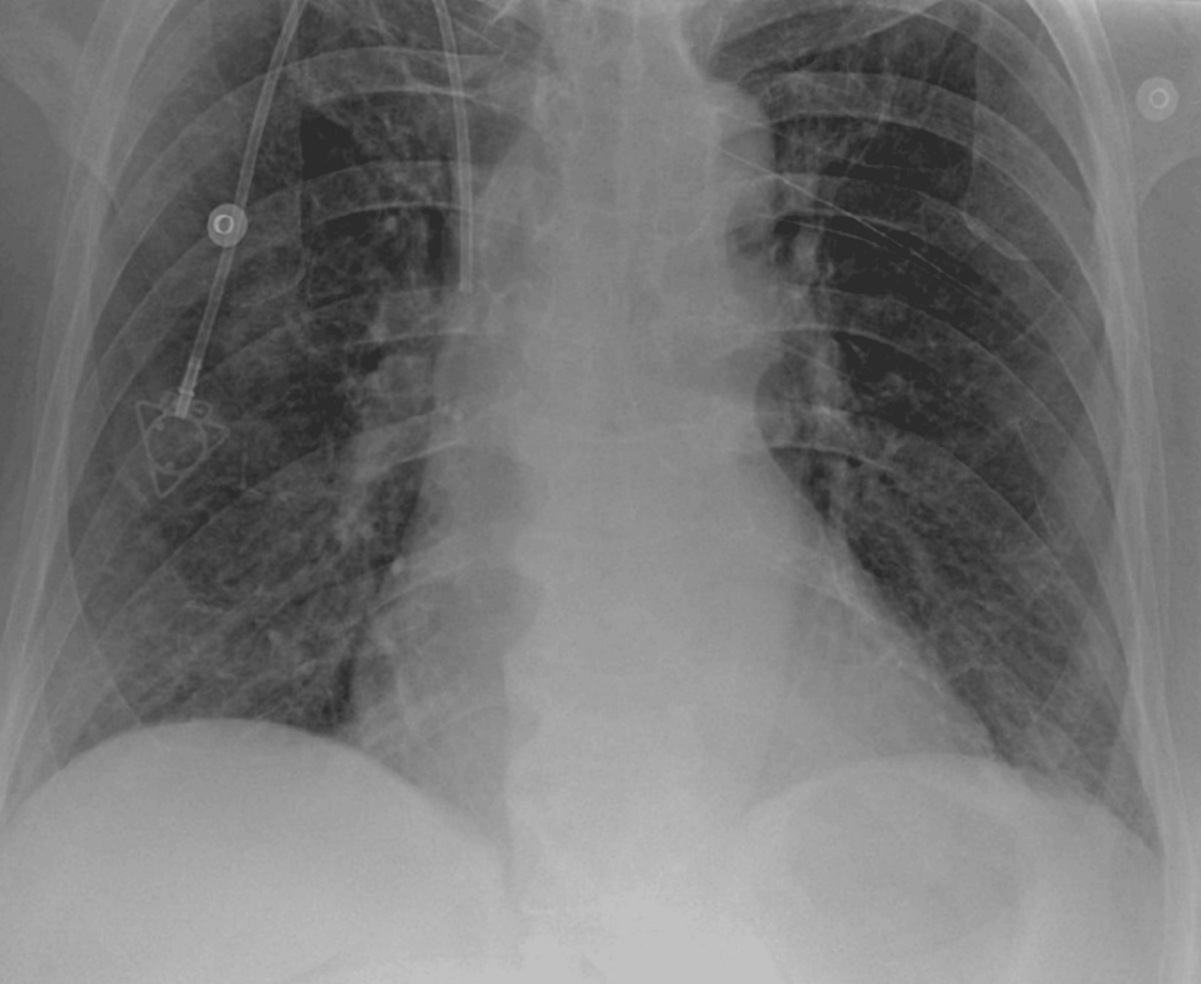
Chest X-ray showing the resolution of pleural effusion following the cessation of imatinib

She was admitted to the hospital and underwent thoracentesis with pleural fluid studies showing zero neutrophils, elevated lactate dehydrogenase (LDH) at 197, pleural fluid protein at 2.2, and serum protein at 4, indicating exudative fluid (Table 1). Cytology from the pleural fluid was negative for malignancy, and cultures were also negative. An echocardiogram showed normal heart function, and the patient exhibited no signs of volume overload on examination. Her symptoms improved after thoracentesis, and she was discharged. Following discharge, the imatinib dose was reduced from 400 mg twice daily to 400 mg once daily. However, she was re-admitted a few weeks later with worsening pleural effusions and required repeat thoracenteses. Imatinib was permanently discontinued, which led to the resolution of her pleural effusions, as shown in Figure 4.

**Table 1:** Laboratory results LDH: lactate dehydrogenase

Parameter	Result	Reference Range
Neutrophils (cell/µL)	0	2500–7000
LDH (U/L)	197	140–280
Pleural fluid protein (g/dL)	2.2	<1.5
Serum protein (g/dL)	4	6–8

She remained on pembrolizumab for metastatic lung cancer. After discontinuing imatinib for GIST, she was switched to sunitinib, but treatment was complicated by severe mucositis, fatigue, and pancytopenia, leading to its eventual discontinuation. For her metastatic lung cancer, she continued pembrolizumab maintenance therapy for a total of 35 cycles before developing a grade 3 rash, necessitating the cessation of pembrolizumab. Both her GIST and metastatic lung cancer have fortunately remained stable off treatment, and she is currently doing well on surveillance.

## Discussion

Imatinib is a TKI primarily used to treat CML and GISTs. It targets and inhibits specific tyrosine kinases, such as BCR-ABL in CML and c-KIT in GISTs, which are crucial for cancer cell proliferation. By binding to the ATP-binding site of these kinases, imatinib blocks their phosphorylation activity, thereby halting the signaling pathways that promote cell growth and survival [5,6]. This targeted inhibition reduces cancer cell growth and induces apoptosis, leading to effective treatment outcomes with fewer side effects compared to conventional chemotherapy.

Pleural effusion refers to the accumulation of excess fluid within the pleural space, the narrow compartment situated between the visceral pleura, which envelops the lungs, and the parietal pleura, which lines the inner surface of the chest wall. It can be classified into exudative and transudative types based on the fluid's characteristics and underlying causes. Exudative effusions are typically caused by conditions that increase capillary permeability, such as infections, malignancies, or inflammatory diseases. Transudative effusions result from systemic factors that alter pressure gradients, like heart failure, liver cirrhosis, or nephrotic syndrome. Diagnosis involves imaging studies, such as chest X-rays or ultrasounds, and analysis of the pleural fluid obtained via thoracentesis [7].

Light's criteria are used to differentiate between exudative and transudative pleural effusions. According to Light's criteria, a pleural effusion is classified as exudative if at least one of the following conditions is met: the pleural fluid protein to serum protein ratio is greater than 0.5, the pleural fluid LDH to serum LDH ratio is greater than 0.6, or the pleural fluid LDH level is more than two-thirds the upper limit of normal serum LDH. If none of these criteria are met, the effusion is classified as transudative [8].

**Figure 5:**
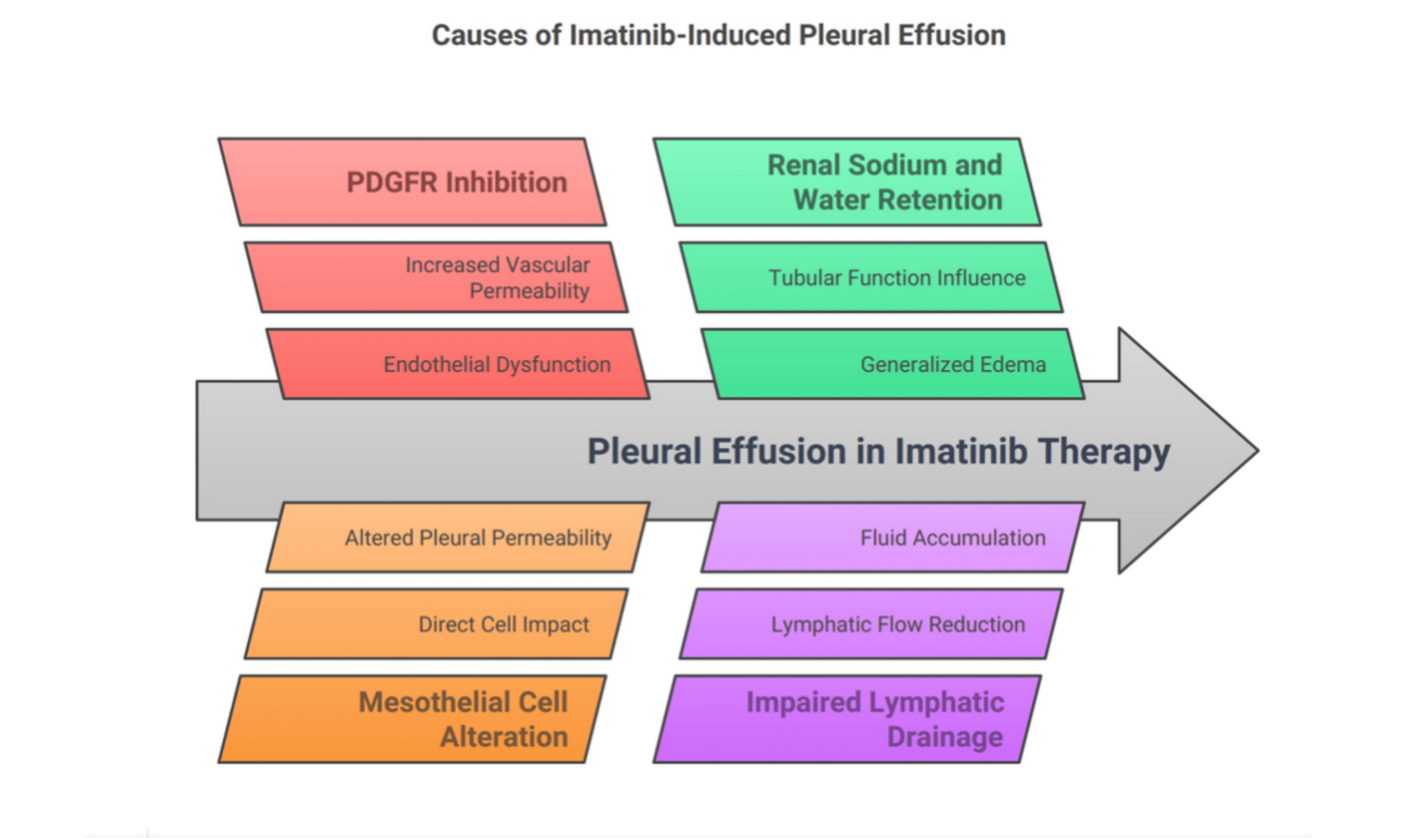
Pathophysiology mechanisms of imatinib-induced pleural effusion PDGFR: platelet-derived growth factor receptor Image Credits: Pranali S. Pachika and Ranadheer R. Dande

One common side effect of imatinib therapy is edema, typically presenting as mild periorbital and lower extremity swelling. However, in rare cases, particularly with higher doses, imatinib can cause significant fluid retention, leading to severe pleural and pericardial effusions [9]. While pleural effusions are more commonly associated with the TKI dasatinib, they are rarely reported with imatinib [10]. Few mechanisms were elucidated on how imatinib causes pleural effusion. It is believed that imatinib-induced pleural effusion is a multifactorial process primarily driven by its inhibition of platelet-derived growth factor receptors (PDGFRs), which play a crucial role in maintaining vascular integrity. PDGFR inhibition leads to endothelial dysfunction and increased vascular permeability, resulting in fluid extravasation into the pleural space [11]. Additionally, imatinib directly affects mesothelial cells, altering pleural permeability and contributing to effusion formation. Renal sodium and water retention further exacerbate fluid accumulation, as imatinib influences renal tubular function, leading to generalized edema, including pleural effusion [12]. Impaired lymphatic drainage has also been implicated, reducing the clearance of pleural fluid. Moreover, hypoalbuminemia observed in some patients on imatinib therapy lowers oncotic pressure, promoting fluid shifts into the pleural space [13]. These mechanisms collectively contribute to the development of pleural effusions in patients receiving imatinib therapy. Figure 5 illustrates the different mechanisms through which imatinib may lead to the development of pleural effusion. Adjusting the imatinib dose can often alleviate the symptoms, as reducing the dose tends to decrease fluid retention and improve the condition [11-13]. However, our patient developed significant bilateral pleural effusions despite taking imatinib only once daily, and ultimately, discontinuing the medication led to an improvement in her condition.

## Conclusions

This case highlights a rare but significant side effect of imatinib therapy, the development of recurrent pleural effusions, which resolved after discontinuing the imatinib. While imatinib is an effective treatment for CML and GISTs, its potential to cause fluid retention, including pleural effusion, should not be overlooked, particularly when other causes are not evident. Clinicians should be vigilant in monitoring for this rare complication, especially in patients receiving higher doses of imatinib, as it may require prompt intervention and discontinuation of the drug to ensure patient well-being. This case highlights the importance of considering imatinib as a possible etiology for unexplained pleural effusions and reinforces the need for careful management and dose adjustments in patients experiencing fluid retention. Ultimately, recognizing and addressing such side effects can improve patient outcomes and prevent unnecessary diagnostic delays.
